# Nailfold videocapillaroscopy in antineutrophil cytoplasmic antibody–associated vasculitis

**DOI:** 10.1186/s13075-023-03227-z

**Published:** 2024-01-02

**Authors:** Megan M. Sullivan, Andy Abril, Nabeel Aslam, Colleen T. Ball, Florentina Berianu

**Affiliations:** 1 Division of Rheumatology, Mayo Clinic School of Graduate Medical Education, Mayo Clinic College of Medicine and Science, 13400 E Shea Boulevard, Scottsdale, AZ 85259 USA; 2 Division of Rheumatology, Mayo Clinic, Jacksonville, FL USA; 3 Division of Nephrology and Hypertension, Mayo Clinic, Jacksonville, FL USA; 4 Division of Clinical Trials and Biostatistics, Mayo Clinic, Jacksonville, FL USA

**Keywords:** Antineutrophil cytoplasmic antibody, Diagnostic imaging, Granulomatosis with polyangiitis, Microscopic polyangiitis, Vasculitis

## Abstract

**Objective:**

Antineutrophil cytoplasmic antibody (ANCA)–associated vasculitis (AAV) is a group of illnesses that cause inflammation and alterations to small vessels in the body. Some of the most common and detrimental manifestations, including alveolar hemorrhage and glomerulonephritis, are caused by this capillary inflammation. We sought to clarify whether patients with AAV would have abnormal nailfold capillaries when evaluated with nailfold videocapillaroscopy.

**Methods:**

Patients with a current diagnosis of AAV and a control group were identified for enrollment. Nailfold videocapillaroscopy images were used for a semiquantitative analysis on capillary density, morphology, dilation, and microhemorrhage after review by 2 rheumatologists. Disease characteristics, occurrence of recent disease flare, and presence of ANCA were recorded.

**Results:**

Thirty-three patients with a diagnosis of AAV and 21 controls were recruited. The AAV group had a median age of 59 and 17 (52%) were women. Granulomatosis with polyangiitis was the most common diagnosis (19 [58%]), followed by eosinophilic granulomatosis with polyangiitis (7 [21%]) and microscopic polyangiitis (7 [21%]). Twenty-seven patients (82%) had positive ANCA tests. After assessment of capillary density, dilation, morphology, microhemorrhages, and disorganization, there were no statistically significant differences between the 2 groups.

**Conclusion:**

There was no evidence of differences in nailfold capillaroscopy abnormalities between those diagnosed with AAV and the control group. While this cohort was relatively small, we did not find a high enough prevalence or specific phenotype of capillary abnormalities that could aid in diagnosis or prognostication of these diseases in the clinical setting.

## Key points

• Our research group questioned whether capillaroscopy would identify abnormalities in those with known ANCA associated vasculitis, which is known to induce capillary inflammation in other organs such as the lungs and kidneys.

• No large differences in capillaroscopy findings were identified between groups.

• Capillaroscopy is unlikely to be of immediate clinical benefit to the practicing rheumatologist.

## Introduction

Antineutrophil cytoplasmic antibody (ANCA)–associated vasculitis (AAV) is a group of rheumatologic illnesses that cause inflammation and alterations to small vessels in the body. It is estimated to have a prevalence of 300 to 421 per 1 million persons [1–3] and consists of granulomatosis with polyangiitis, microscopic polyangiitis, and eosinophilic granulomatosis with polyangiitis. The hallmark clinical manifestations of these diseases, including glomerulonephritis and diffuse alveolar hemorrhage, are caused by capillary damage to different organ systems. This organ damage is prevalent, with end-stage renal disease occurring in up to 25% of patients with AAV [4]. Current monitoring strategies require close follow-up and oftentimes expensive laboratory and imaging assessments.

Given the known capillary inflammation central to the pathophysiology of small-vessel vasculitis, several small studies have used a technique known as *capillaroscopy* to evaluate patients [5–9]. Capillaroscopy consists of placing a magnifying scope on the nailfold to assess for capillary abnormalities, such as microhemorrhages, changes in normal vessel architecture, and decreases in capillary density. Abnormal capillaroscopy findings were reported in patients with granulomatosis with polyangiitis, immunoglobulin A vasculitis, and cryoglobulinemic vasculitis [5–7, 9]. The abnormalities in these studies were statistically significant when compared with healthy controls and included nailfold hemorrhages, changes in capillary density, increased capillary tortuosity, bushy capillaries, and loss of normal architecture. One study of 29 patients with cryoglobulinemic vasculitis reported further statistical correlation of capillaroscopy abnormalities and renal disease secondary to vasculitis [8].

Nailfold capillaroscopy has advanced to include nailfold videocapillaroscopy (NVC) which provides higher-quality images. It is inexpensive, quick, and provides objective data with high interreader reliability among users [10, 11]. NVC is used in rheumatologic clinics due to its prognostic and diagnostic benefits for patients with Raynaud disease or early mixed connective tissue disease. In these patients, the vascular abnormalities seen on NVC are indicative of vascular damage occurring in other organs in the body, such as pulmonary arterial hypertension seen in patients with scleroderma. Furthermore, defined NVC phenotypes identified for scleroderma and dermatomyositis can be useful for prognostication and diagnosis [12–18]. It is unclear whether this can be used in a similar fashion in AAV. The primary objective of this study was to compare the proportion of patients with NVC abnormalities in patients with AAV compared to healthy controls.

## Methods

### Study population and data collection

Patients with a diagnosis consistent with small-vessel vasculitis and control patients without a diagnosis of small-vessel vasculitis were recruited from our rheumatology clinic. Recorded baseline information included clinical diagnosis, history of vasculitis-related organ involvement, medication use, and pertinent laboratory results, including ANCA serologies. Current rituximab use was recorded if the patient had received a rituximab infusion in the preceding 6 months. Patients were excluded if they had a history of Raynaud phenomenon or disease, scleroderma, antiphospholipid syndrome, type 2 diabetes, dialysis dependence, bleeding disorder, or diagnosis of psoriasis. Recent hand trauma or manicure (within 2 weeks of assessment), routine use of vibratory tools, and exposure to toluene or benzoyl were additional exclusion criteria.

### Nailfold videocapillaroscopy

Capillaroscopy was performed in a room with air temperature between 22° and 25° Celsius to prevent vasoconstriction, with the patient seated in the examination room for approximately 10 to 15 min prior to examination. Immersion oil or cedarwood oil was applied to the patient’s nailfolds and a minimum of 2 images per nailfold was saved for 8 digits (excluding thumbs) per patient. For inclusion, 80% of the images had to be moderate to high quality, with uninterpretable images taken out of the scoring. Capillaroscopy parameters included density, dimension, morphology, and hemorrhage. Density was defined as abnormal if there were fewer than 9 capillaries per 1-mm field. An abnormal dimension was greater than 20 μm, with 20 to 49 μm defined as enlarged and greater than 50 μm as giant. Morphology was considered abnormal if capillaries did not have the characteristic hairpin shape at the end. Abnormal morphology was categorized into diagnoses of bushy capillaries or tortuosity (not recorded). Presence of micro- or macrohemorrhages were considered abnormal. A semiquantitative scoring system was used as previously outlined in the literature [19] with possible scores of 0 to 3 in each category, higher scores indicating higher prevalence of abnormalities.

### Statistical methods

Based on prior literature, it was anticipated that between 50 and 80% of patients with AAV would have an abnormal NVC. We calculated that a sample size of at least 20 patients per group would achieve 80% power at the 5% significance level to detect a difference in proportions between groups of 0.45 (e.g., 50% vs 5% with abnormal NVC when comparing AAV vs controls) using a 2-sided Fisher exact test allowing for up to 15% of patients to have missing data or problems with measurements.

Continuous data were summarized with sample median and interquartile range (IQR). Categorical data were presented as number and percentage of patients. For the primary analysis, we compared the proportion of patients with any abnormal NVC findings between the patients diagnosed with AAV and the control group using Fisher exact test. As a secondary analysis, we used linear regression models to estimate the difference in NVC scores between those with AAV and the control group adjusting for age, sex, and smoking history; corresponding 95% CIs and *P* values were reported. All *P* values are 2-sided without adjustment for multiple testing. *P* values less than.05 were considered statistically significant. R, version 4.1.2 (R Foundation for Statistical Computing), was used for analysis.

## Results

**Fig. 1:**
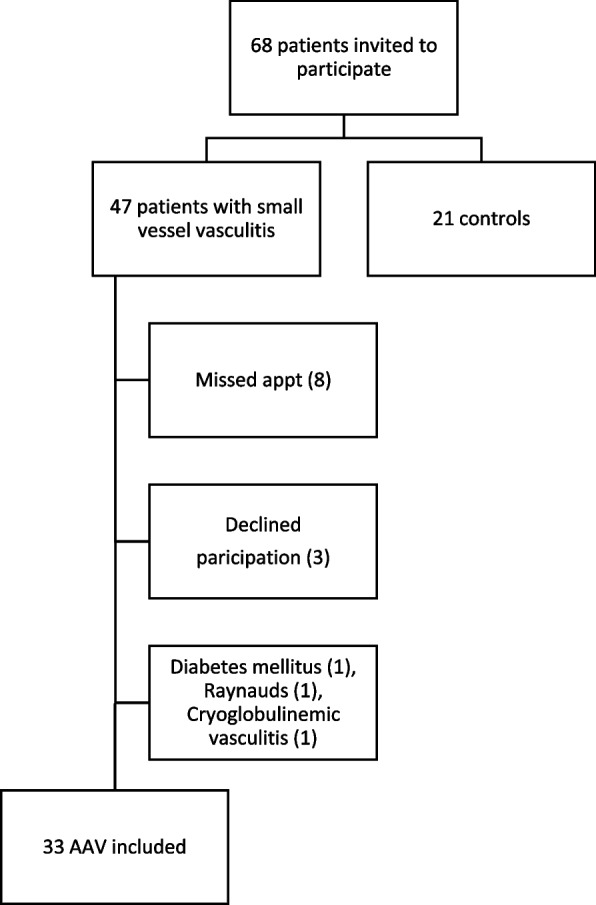
Patient enrollment. AAV indicates antineutrophil cytoplasmic antibody–associated vasculitis

Forty-seven patients with a diagnosis consistent with small-vessel vasculitis were recruited from our rheumatology clinic along with 21 control patients (Fig. 1). Several patients did not undergo capillaroscopy due to meeting exclusion criteria, not presenting for the appointment, or declining participation. Thirty-four patients with small-vessel vasculitis and 21 control patients without AAV underwent capillaroscopy. We excluded 1 patient with cryoglobulinemic vasculitis for a total of 33 patients with AAV included in our analysis.

Baseline characteristics are shown in Table 1. Median age was 9 years older in the AAV group compared to the control group (median 59 years vs 50 years). ANCA was positive in 27 patients (82%) in the AAV group; specific ANCA patterns are noted in Table 1. The most common immunosuppressive medication used in the AAV group was rituximab (22 [67%]), followed by methotrexate (6 [18%]), azathioprine (3 [9%]), and oral cyclophosphamide (1 [3%]). Prednisone use was recorded at the time of capillaroscopy for 15 patients (45%). Only 1 patient was taking doses greater than 20 mg/day. Average C-reactive protein was 10.58 mg/L.

**Table 1:** Baseline characteristics

Characteristic	AAV (*n* = 33)	Control (*n* = 21)
Age, median (IQR), years	59 (48, 67)	50 (39, 56)
Ethnicity, no. (%)		
Hispanic or Latino	1 (3.0)	0 (0.0)
Not Hispanic or Latino	31 (93.9)	21 (100.0)
Unknown or not reported	1 (3.0)	0 (0.0)
Sex, no. (%)		
Female	17 (51.5)	14 (66.7)
History of smoking, no. (%)	11 (33.3)	7 (33.3)
Diagnosis, no. (%)		
EGPA	7 (21.2)	
GPA	19 (57.6)	
MPA	7 (21.2)	
ANCA positivity, no. (%)	27 (81.8)	
c-ANCA/PR3	15 (45.5)	
p-ANCA/MPO	9 (27.3)	
p-ANCA/PR3	2 (6.1)	
MPO	1 (3.0)	

*Abbreviations*: *AAV* antineutrophil cytoplasmic antibody–associated vasculitis, *ANCA* antineutrophil cytoplasmic antibodies, *c-ANCA* cytoplasmic antineutrophil cytoplasmic antibodies, *EGPA* eosinophilic granulomatosis with polyangiitis, *GPA* granulomatosis with polyangiitis, *MPA* microscopic polyangiitis, *MPO* myeloperoxidase, *p-ANCA* perinuclear antineutrophil cytoplasmic antibodies, *PR3* proteinase 3

For the primary analysis, we found no difference in the proportion of patients with abnormal NVC between those with AAV (18/33 [55%]) and those in the control group (11/20 [55%]; *P* = 1.00). In secondary analysis, we did not find differences in NVC scores between the AAV and control groups for capillary density, microhemorrhage, capillary dilation, giant capillaries, ramification, and disorganization (Table 2).

**Table 2:** Nailfold videocapillaroscopy results

	**AAV (*n* = 33)**^a^	**Control (*n* = 21)**^a^	***P* value**^b^
Nailfold videocapillaroscopy (primary)	18 (54.5)	11 (55.0)^c^	1.00
Capillaroscopy density	3 (90.9)	0 (0.0)	.28
Dilated capillaries	7 (21.2)	4 (19.0)	1.00
Giant capillaries	0 (0.0)	0 (0.0)	1.00
Microhemorrhages	13 (39.4)	10 (47.6)	.58
Capillary ramification	4 (12.1)	0 (0.0)	.15
Capillary disorganization	2 (6.1)	0 (0.0)	.52

*Abbreviation*: *AAV* antineutrophil cytoplasmic antibody–associated vasculitis

^a^The number (%) of patients with abnormal nailfold videocapillaroscopy findings is shown separately for each patient group. An abnormal finding was defined as a score greater than 0 (range, 0–3)

^b^Fisher exact test

^c^Capillaroscopy density was not available for 1 participant in the control group; that participant was not included in the denominator for the primary outcome

**Fig. 2:**
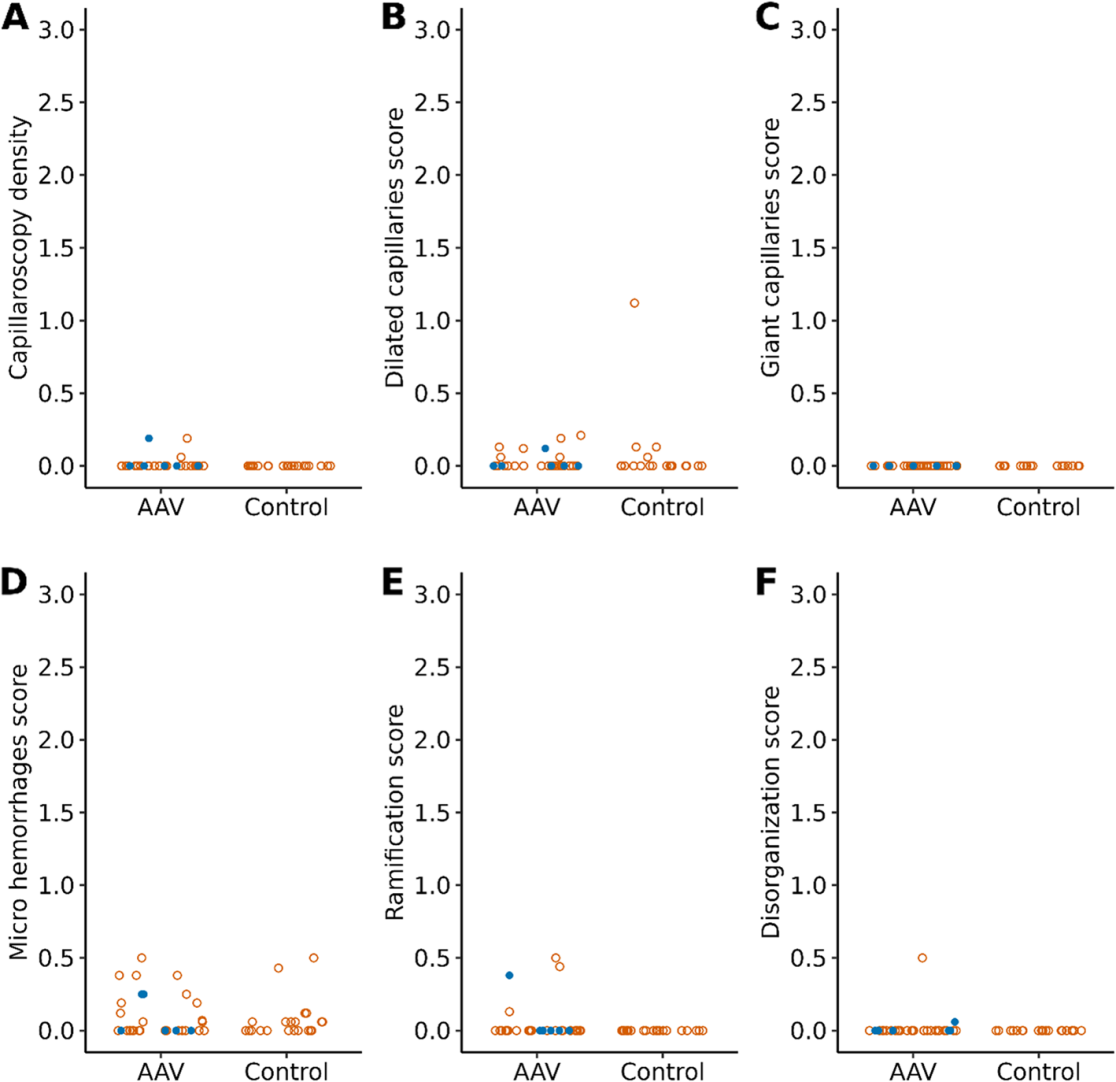
Jitter plot of nailfold videocapillaroscopy scores. **A** Capillaroscopy density. **B** Dilated capillaries score. **C** Giant capillaries score. **D** Microhemorrhages score. **E** Ramification score. **F** Disorganization score. Blue dots represent patients who had a flare within 3 months of capillaroscopy. Due to high frequency of 0 scores, points were jittered horizontally to minimize overlap of points. AAV indicates antineutrophil cytoplasmic antibody–associated vasculitis

**Fig. 3:**
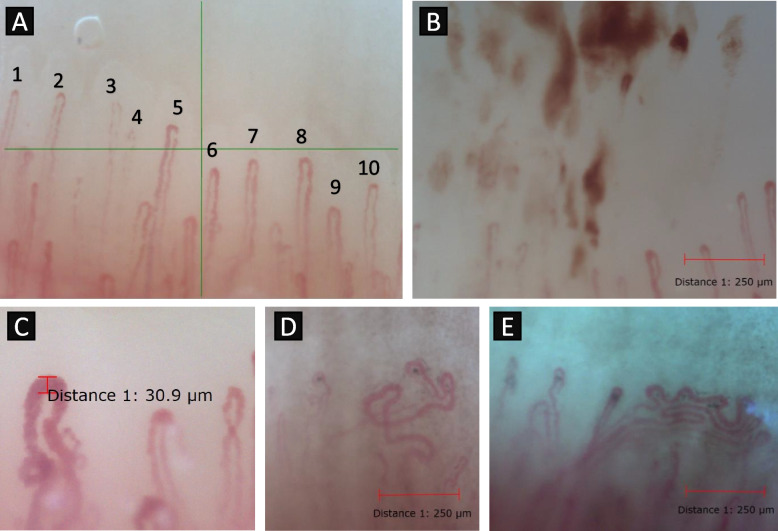
Representative nailfold videocapillarosocpy images. **A** Example of a capillary count within 1 mm. **B** Microhemorrhage in a patient with granulomatosis with polyangiitis. **C** Capillary dilation in a control patient. **D**, **E** Bushy capillary formation in a patient with seronegative granulomatosis with polyangiitis

Figure 2 shows the distribution of scores for individual components of NVC for each group. A model of capillary density calculation and examples of capillary changes found in our study are shown in Fig. 3.

## Discussion

Capillaroscopy has gained traction in rheumatology for its known correlation to systemic sclerosis and dermatomyositis, which have considerable microvasculature change. In the setting of systemic sclerosis, capillaroscopy abnormalities have a distinct enough phenotype to help aid in diagnosis. Endovascular dysfunction is felt to occur early in the disease with a reduction in nitric oxide, increased cell adhesion molecules for inflammatory cell chemotaxis, and eventually a fibroproliferative process [20]. In dermatomyositis, a different capillary pattern is seen with neoangiogenic change (e.g., bushy capillaries, loss of architecture). The microvascular change is felt to be immune mediated, perhaps due to increased type 1 interferon and complement deposition [21]. Interestingly, capillaroscopy changes are reversible with therapy in dermatomyositis, indicating it could potentially be used to monitor disease activity [16].

AAV is a group of illnesses characterized by inflammation of the capillary beds that preferentially affect the skin through vascular rashes, lungs through diffuse alveolar hemorrhage, and kidneys through glomerulonephritis. Studies have shown that this is a pauci-immune process without immunoglobulin or complement deposition. There is a loss of tolerance that allows for antibody development to myeloperoxidase and proteinase 3. These ANCA then stimulate neutrophils to further the inflammatory environment within the capillary beds [22]. Given the multiorgan microvascular inflammation that occurs in these conditions, our study aimed to assess for capillary changes in the nailfold using capillaroscopy, a widely accessible clinical tool.

A recent publication by Matsuda et al. [23] compared 51 patients with AAV with healthy controls. They reported AAV had statistically relevant changes in all components of the capillaroscopic examination, which is in contrast to our own findings. Several phenotypes were described, including a microangiopathic pattern (70.6%), scleroderma pattern (7.8%), and normal examination (21.6%). All patients in their cohort with AAV had active disease and were hospitalized, which is a notable difference from our own cohort which was enrolled from clinic. It is possible that these findings correlate more with severe disease, and their article did suggest improvement in the NVC findings posttreatment [23]. Another consideration would be that the critically ill or hospitalized patient may be more likely to have nonspecific capillaroscopy findings such as microhemorrhage due to other contributing factors (e.g., hypercoagulability due to critical illness, low-dose heparin for deep vein thrombosis prevention, microvascular change from hypertension and hyperglycemia). This could be addressed with additional studies accounting for the confounding data that having an outpatient control and inpatient disease cohort could introduce.

Frequent capillary abnormalities have been reported, usually at low levels, in the general population [24]. Similarly, our control group had some higher scores than expected: 1 patient with capillary dilation and 2 with frequent microhemorrhages. Known patient history may explain some abnormality (e.g., 1 patient with multiple microhemorrhages cleans houses with repeated scrubbing using the nails); however, unknown variables may have also altered the examinations. Other illnesses that affect the vasculature have been noted to cause change on capillaroscopy, including diabetes mellitus and dialysis dependence. Bleeding disorders and recent trauma to the nailfold can also result in abnormal findings. Overall, this highlights that mild capillary abnormalities can be seen in typical patient populations and are not exclusive to rheumatic disease.

Our study has several limitations. There was a lack of blinding for the capillaroscopy procedure; however, this would have been expected to skew the results to overestimate differences. Furthermore, while all patients in the AAV group had a diagnosis of AAV, some were in clinical remission at the time of review and only 1 in 5 had experienced a recent flare. Ideally, the study would have included new incidental cases with active disease. However, we do note that abnormalities were minimal even in patients with recent disease activity. For practical clinical use, we feel that patients would have to have widely prevalent nailfold capillary change or at least a strong phenotype with active disease; however, neither of these criteria were met. The significant prevalence of abnormal capillaroscopy findings and specific phenotype are the reasons capillaroscopy has been found to be useful in dermatomyositis and systemic sclerosis. Further studies are still needed to clarify whether there are meaningful subgroups withing these vasculitides that would benefit from capillaroscopy.

## Conclusion

We compared the prevalence of abnormal NVC findings between patients with a diagnosis of AAV to control patients without AAV and did not find a significant difference in the NVC scores. We found no indication of a high enough prevalence of NVC abnormalities or strong enough phenotype to be useful in the clinic setting for diagnosis or prognostication of AAV.

## Data Availability

The datasets used and/or analyzed during the current study are available from the corresponding author on reasonable request.

## Abbreviations

ANCA: Anti-neutrophilic cytoplasmic antibody
AAV: ANCA-associated vasculitis
NVC: Nailfold videocapillaroscopy
